# Energy costs and benefits of locomotion and feeding in site-attached damselfish

**DOI:** 10.1242/jeb.251164

**Published:** 2025-10-13

**Authors:** Kota Ishikawa, Heng Wu, Satoshi Mitarai, Amatzia Genin

**Affiliations:** ^1^ Marine Biophysics Unit, Okinawa Institute of Science and Technology Graduate University, Onna, Okinawa 904-0495, Japan; ^2^ The Interuniversity Institute for Marine Sciences in Eilat and Department of Ecology, Evolution and Behavior, The Hebrew University of Jerusalem, Eilat 88103, Israel

**Keywords:** Oxygen consumption, Foraging, Flow, Fish swimming, Respirometry, Habitat selection

## Abstract

Energetic cost–benefit balance provides valuable information on the environmental tolerances and distributions of animals. In aquatic environments, flow is a fundamental factor owing to its effects on locomotion and foraging. Energetic trade-offs have been well studied in river fishes, but remain understudied in coral reef fishes. Here, we assessed energy balance of the coral reef damselfish (*Chromis viridis*) by measuring its oxygen consumption and feeding rate. To accurately estimate energy costs during feeding maneuvers, oxygen consumption during feeding was estimated by the video-based dynamic body acceleration method. Our results indicate that the energetically favorable range of flow speed was 17–29 cm s^−1^, comprising approximately 25% of the flow speed in the fish habitat. By simulating lower prey densities, we also found critical combinations of prey density and flow speed at an energy balance. Our findings provide insights into adaptation and habitat use of site-attached fish in coral reefs.

## Teaser

**Summary:** An empirical cost–benefit balance for a site-attached damselfish in coral reefs provides insights into habitat selection and environmental quality assessment.

## INTRODUCTION

Net energy gain is an important concept in fish ecology, defined as energy intake by feeding minus the cost of swimming and maintenance; thus, net energy is available for growth and reproduction (Fausch, 1984; Hill and Grossman, 1993; Piccolo et al., 2014). By assessing net energy gain as a function of environmental parameters, many cost–benefit models have been developed to estimate habitat selection of river fish that feed on drifting prey in flowing water. This feeding style results in energy optimization, as fish select habitats to maximize energy gain and minimize energy expenditure (Hill and Grossman, 1993; Piccolo et al., 2014). In these models, flow speed is a dominant parameter because it affects both drifting prey flux and swimming cost. Since Fausch (1984) developed the first quantitative energy cost–benefit model, similar models have been used to assess habitat selection and environmental quality (reviewed in Piccolo et al., 2014).

Flows play a significant role in shaping species distribution and community structure in coral reefs, through their effects on behavior and morphology (Binning and Roche, 2015; Fulton and Bellwood, 2005; Fulton et al., 2005; Korsmeyer et al., 2002). For example, body-caudal fin swimmers use lateral undulation of the body and caudal fin to create thrust, which provides greater power for sustained swimming in fast flows, whereas median-paired fin swimmers use median or paired fin propulsion, which provides better maneuverability in complex environments (Blake, 2004; Webb, 1984). Although most fish are body-caudal fin swimmers, more than 60% of fish in coral reefs are pectoral fin swimmers (Fulton and Bellwood, 2005), reflecting adaptations to the complex geometry and flow regimes in coral reefs. To understand how these morphological and functional differences help fish energetically to live in different flow environments, comparative studies have examined oxygen consumption of fish with different swimming modes and morphology and have provided significant understanding of suitable habitats for different types of fish (Korsmeyer et al., 2002; Marcoux and Korsmeyer, 2019; Schakmann and Korsmeyer, 2023). However, these studies often overlook effects of flow on energy gain, despite its substantial influence on prey flux and foraging (Clarke et al., 2009; Genin et al., 2024; Ishikawa et al., 2022; Kiflawi and Genin, 1997). Hence, examining how flows modify both energy gain and expenditure is essential for a more accurate understanding of adaptation and habitat selection of site-attached coral reef fishes.

In previous cost–benefit studies, energy cost has usually been estimated assuming fish engage only in steady swimming because it is challenging to balance enough volume needed for the fish's free-ranging behaviors and limited volume needed for respirometry. However, behaviors that involve rapid changes in speed and direction are expected to require at least two to three times more energy than steady swimming (Hughes and Kelly, 1996; Krohn and Boisdair, 1994; Schakmann et al., 2020; Tang et al., 2000). Therefore, the cost of feeding maneuvers should be integrated in estimation of the cost–benefit balance. Recent advances in automatic body tracking and acceleration-based energy cost estimation using videos have enabled quantification of oxygen consumption rates during free-ranging behaviors of planktivorous fish (Ishikawa et al., 2025). Using this technique, the energy cost of feeding behavior of planktivorous fish can be quantified to understand energy cost and benefit more accurately.

In this study, our goal was to quantify energy cost and benefit of a site-attached damselfish (*Chromis viridis*) inhabiting coral reefs worldwide. To understand its energy cost and gain during feeding, we estimated oxygen consumption rates and feeding rates of the fish in a flume across a range of flow speeds representative of their natural habitat. The result was then used to assess the optimal conditions and limitations of the fish distribution.

## MATERIALS AND METHODS

### Flow measurements in the field

Field observations were conducted in the natural habitat of the blue-green chromis (*Chromis viridis* Cuvier 1830) in Onna, Okinawa, Japan (26°30′27N, 127°51′23″E). Our near-shore study site was approximately 3 m deep and strewn with *Acropora*-bearing knolls that provided shelter for groups of *C. viridis*. In the habitat area, the maximum range of tides was approximately 2 m. An acoustic Doppler velocimeter (ADV; Vector, Nortek) was deployed at this site from 27 September to 27 October 2022, set to measure velocities at 32 Hz. The ADV measured a certain volume ∼50 cm from a patch reef (220 cm above the bottom) to match the volume observed in the feeding fish. Every 30 min, the ADV initiated a 10-min measurement burst. Velocity data were screened for low-beam correlations (<60%) and missing data were interpolated linearly. Mean flow speeds were computed by averaging flow velocities along all three axes for each 10-min burst (Gross and Nowell, 1983). Measurements were also conducted at a sandy site with scattered patches of rocks and coral, 16 m deep, where no *C. viridis* were found (26°30′39″N, 127°52′44″E) from 14 July to 14 August 2022 to compare flow speeds.

### Study fish

Five *C. viridis* originated from Okinawa, Japan, were purchased from Aqua Planning Co., Ltd, and designated A to E, with body masses of 6.59, 6.58, 5.17, 8.87 and 9.32 g, respectively. The fish were kept in a holding tank and fed with brine shrimp (*Artemia salina*) nauplii *ad libitum*. All experiments were conducted with approval from the Animal Care and Use Committee at Okinawa Institute of Science and Technology Graduate University.

### Experiment

To understand energy gain at a specific flow speed, feeding rates at different flow speeds were examined. To ensure that fish had enough volume in which to swim during foraging, experiments were conducted in a custom-made flume, described in Ishikawa et al. (2022), with some modifications. Briefly, the flume was a horizontal recirculating open channel with a rectangular cross section. The test section was 150 cm long, 30 cm wide and 18 cm high. At the inlet and outlet of the test section were flow straighteners, a contraction and a diffuser with a slope of 0.1. The water temperature was maintained at 25.5±0.5°C. Two lights (Mitras lightbar 60, GHL, Germany) were placed above the center of the experimental section and turned on between 07:00 h and 19:00 h (12 h:12 h light:dark). Experiments were conducted between 15 May and 17 July 2023. We conducted the experiments around the same period to eliminate possible seasonal fluctuations of basal metabolic conditions (Beamish, 1964).

Feeding experiments were conducted following Ishikawa et al. (2022) at flow speeds of 5 to 30 cm s^−1^ at intervals of 5 cm s^−1^, which covers most of flow speed observed in their habitat. We defined one water cycle as the time for full circulation of water in the recirculating flume based on the mean flow speed. Before each trial, we fitted a custom-made 100-µm plankton net with a square frame tightly across the flume and ran the water at the specified flow speed for ≥30 min to remove particles from the flume for ≥2 cycles. Given the 1.2 m^3^ volume of the flume, individual live nauplii were manually counted to obtain a prey density of 1000 m^−3^ in each trial. Prey were gradually released downstream of the center section during one water cycle to achieve a nearly homogeneous prey distribution. One water cycle was 270, 135, 90, 71, 56 and 46 s at flow speed of 5, 10, 15, 20, 25 and 30 cm s^−1^, respectively. After the first prey item appeared in the test section and fish started feeding, we started a trial and recorded a video at 50 frames s^−1^ with 4 K resolution for one water cycle of the flume using an EOS R6 Mark II Canon camera. Two replicates at each flow speed were conducted for five individuals. Six trials per day, one per each flow speed with a randomized order, were conducted and the second replicates were carried out the next day.

Using the video recordings, we counted the number of successful strikes, defined as open-mouthed lunges in which we saw prey items enter fish mouths. Then, we divided the number of successful strikes by the time required for one water cycle to obtain the feeding rate (min^−1^). Because we only observed five occasions (out of 14,048 strike motions) that fish missed prey during the whole experimental series, the success rate was nearly 100%. The relationship between mean feeding rates and flow speed was approximated with the Lagrange polynomial.

To understand energy cost during feeding, we used the video-based dynamic body acceleration (DBA) method (Ishikawa et al., 2025). We recorded fish behavior during feeding experiment using cameras (acA2000-165uc-Basler ace, Basler) at 90 frames s^−1^ with a resolution of 1920×1080 pixels. Two cameras with 25 mm lenses (25 mm C Series Fixed Focal Length Lens, Edmund Optics) were positioned above and on the side of the flume. To obtain vectorial dynamic body acceleration (VeDBA), the videos were analyzed following Ishikawa et al. (2025). In short, an eye of each fish, as in Ishikawa et al. (2025), was tracked using the Python package DeepLabCut (Mathis et al., 2018; Nath et al., 2019). After *P*-value cut, Hampel filtering and mean filtering, 2D coordinates from the videos were transformed into 3D coordinates using direct linear transformation (MATLAB package easyWand5; Hartley and Zisserman, 2004; Theriault et al., 2014). Finally, using the eye position time series in 3D, we employed the second-order forward finite difference method to obtain instantaneous acceleration in each axis (*a_x_*, *a_y_*, *a_z_*; the *x*-axis as the streamwise direction, the *y*-axis as the lateral direction and the *z*-axis as perpendicular to the bottom) and computed VeDBA as:
(1)

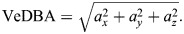
VeDBA values between 0.001 and 1 ***g*** (1 ***g*** as the Earth's gravitational acceleration, 9.8 m s^−2^) were then used to compute mean VeDBA for each trial. Because individuals used in our experiment were identical to those in Ishikawa et al. (2025), we used the generalized linear mixed model between net cost of swimming and DBA (fig. 1D in Ishikawa et al., 2025) to estimate net cost of swimming during feeding in the flume. Standard metabolic rate for each individual, estimated in Ishikawa et al. (2025), was then added back to obtain fish oxygen consumption rates during feeding.

### Energy cost and energy gain

From the oxygen consumption rate, *Ṁ*_O_2__ (mg O_2_ kg^−1^ h^−1^), energy cost per hour (J h^−1^) at a given flow speed was estimated as:
(2)

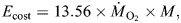
where *M* is the mass of fish (kg), and a conversion rate of 1 mg O_2_=13.56 J was applied (Elliott and Davison, 1975). *E*_cost_ was obtained using *Ṁ*_O_2__ during both feeding and steady swimming. The relationship between *Ṁ*_O_2__ and flow speed during steady swimming (*Ṁ*_O_2__=152.15+0.47*U*^1.76^) was obtained from Ishikawa et al. (2025). In contrast, energy gain per hour at a given flow speed was estimated from the feeding rate. The energy gain per hour (J h^−1^) was computed as:
(3)

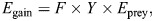
where *F* is the feeding rate (h^−1^), *Y* is a constant describing the energy yield from prey, accounting for digestion and excretion of fish, which was 0.68 (Hill and Grossman, 1993), and *E*_prey_ is the energy content (J) of a copepod, which is a major food source of *C. viridis* (Hiatt and Strasburg, 1960). Assuming copepods are 1.0 mm (Noda et al., 1992), the dry mass of each individual is 16.21 µg (Pearre, 1980). Based on their energy value per dry mass (11.39 J mg^−1^ DM; Chen et al., 2019), *E*_prey_ was 0.18 J.

Assuming 12 h of diurnal feeding and 12 h of nocturnal non-foraging intervals, net energy gain (*E*_net_) was estimated by subtracting *E*_cost_ of 12 h of foraging and 12 h of steady swimming from 12 h of *E*_gain_. As a function of flow speed, *E*_net_ was estimated using the power function of *Ṁ*_O_2__ during steady swimming (fig. 1A in Ishikawa et al., 2025) and the Lagrange polynomial of *Ṁ*_O_2__ and *F* during feeding. By computing the zero-crossing flow speed of the first derivative of *E*_net_, we estimated the flow speed at which fish maximize *E*_net_, in the range of flow speeds tested (5–30 cm s^−1^). The maximum *E*_net_ was limited to 1 to determine the relative net energy gain. An arbitrary threshold of 0.9 was used to identify a range of flow speeds at which fish are able to acquire energy efficiently (Hill and Grossman, 1993).

## RESULTS AND DISCUSSION

### Feeding rates and oxygen consumption rates

**Fig. 1.**
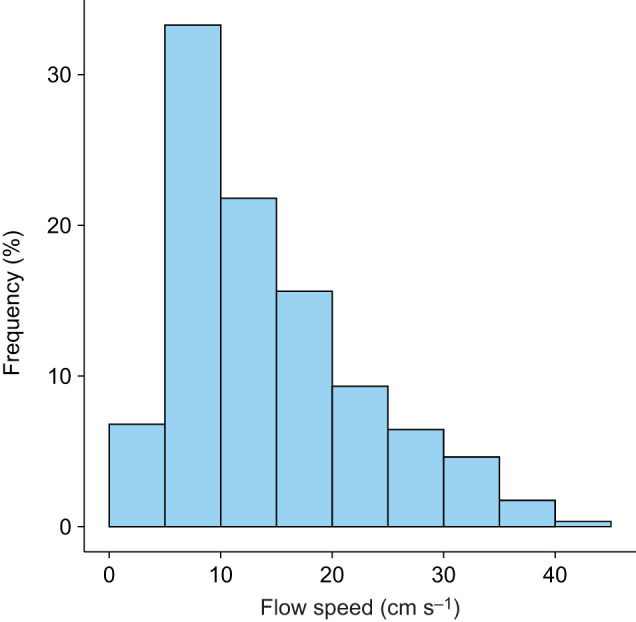
**Frequency distribution of flow speeds in a habitat of *Chromis* *viridis*.** Flow speed was measured by acoustic Doppler velocimeter (ADV) for 10 min every 30 min and averaged over each 10 min measurement. Measurements were conducted between 27 September and 27 October 2022.

**Fig. 2.**
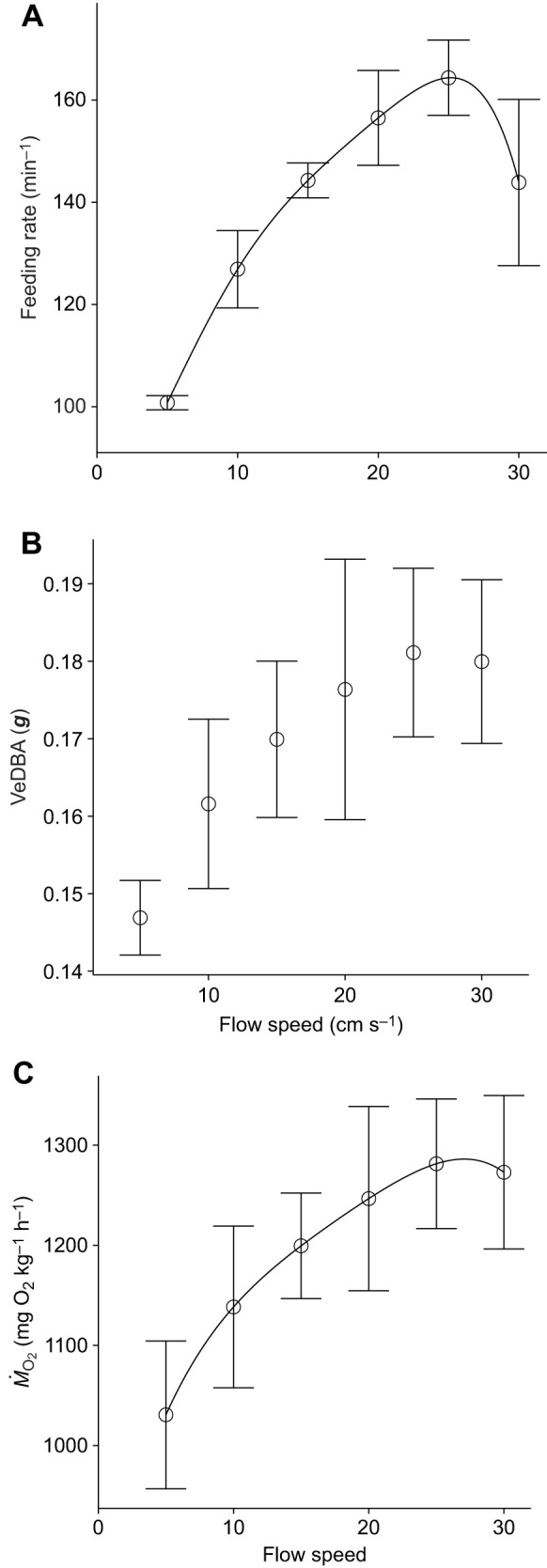
**Effects of flow speed on feeding rate, vectorial dynamic body acceleration (VeDBA) and oxygen consumption rate (*Ṁ*_O_2__).** (A) Feeding rate increased at flow speeds up to 25 cm s^−1^ and decreased at 30 cm s^−1^. (B) VeDBA increased as flow speed increased. (C) *Ṁ*_O_2__, estimated using the video-based DBA method, also increased as flow speed increased. Data points are means±s.d. among individuals (*n*=5). The solid lines are the approximation by the Lagrange polynomial.

Our field flow measurement showed that the mean and maximum flow speeds of all the 10-min averages were 13.9 and 41.5 cm s^−1^, respectively. During the observation, the species spent 86.4% of the time at 5–30 cm s^−1^ (Fig. 1). Based on this field observation, we first examined effects of flow speed (5–30 cm s^−1^) on feeding rate to estimate energy benefit. The feeding rate of *C. viridis* increased at a decelerating rate up to 25 cm s^−1^ and decreased at 30 cm s^−1^ (Fig. 2A). This result corresponds to previous studies on feeding rates of reef fish, showing a unimodal curve in response to flow speed, where feeding increases at slow flows, remains constant at moderate flows, and decreases at faster flows because of the balance between increasing prey flux and decreasing feeding volume (Clarke et al., 2009; Ishikawa et al., 2022; Kiflawi and Genin, 1997). Although the maximum feeding rate occurred at <15 cm s^−1^ in *Dascyllus marginatus* and *C. viridis* in a flume (Kiflawi and Genin, 1997), it occurred at approximately 25 cm s^−1^ in our study. Beside interspecific differences, this difference may be associated with flow speed in natural habitats. Mean flow speeds observed in habitats of *D. marginatus* and *C. viridis* at shallow reefs in the Red Sea are around 5–10 cm s^−1^ (Genin and Paldor, 1998; Monismith and Genin, 2004; Reidenbach et al., 2006), much slower than those in the habitat of *C. viridis* in Okinawa (Fig. 1). Even within the same species living in two different flow regimes around an island, fish show morphological and physiological variations that are suitable for each habitat (Binning et al., 2014), suggesting that long-term exposure to different flows may have caused evolutionary adaptation of the same species to each environment. For river fish, reduction in feeding rate at fast flow speed is associated with reduced success (Hill and Grossman, 1993; Piccolo et al., 2008). However, *C. viridis* captured almost all nauplii they struck in our experiment, suggesting that their maneuverability as a median-paired fin swimmer enables precise body control even at fast flows.

We then examined effects of flow speed (5–30 cm s^−1^) on VeDBA to estimate energy cost during feeding. VeDBA increased as flow speed increased (Fig. 2B). This increase was possibly caused by enhanced maneuvering behaviors, accompanied with enhanced acceleration and deceleration (Krohn and Boisdair, 1994; Tang et al., 2000). Indeed, as we also observed a similar response of feeding rates to flow speed, changes in VeDBA can be partly attributed to the increased number of strike motions (Fig. 2A,B). Using the generalized linear mixed model that describes the relationship between oxygen consumption rates and VeDBA for this set of fish (Ishikawa et al., 2025), we further estimated oxygen consumption rates from the obtained VeDBA. Fish during feeding increased oxygen consumption rate as flow speed increased, and consumed 3.8–6.8 times more oxygen than during steady swimming at each flow speed (Fig. 2C; Ishikawa et al., 2025). Energy costs that involve changes in speed and direction, such as turnings, rapid acceleration and deceleration, were empirically suggested to be approximately 2-fold (Schakmann et al., 2020), 6-fold (Krohn and Boisdair, 1994) or 8-fold (Tang et al., 2000) higher than those of steady swimming. Using a hydrodynamic model, such maneuvers are estimated to have a cost 2.6–10 times higher than that of steady swimming (Hughes and Kelly, 1996). It is worth noting that extrapolation of the calibration curve – extending to approximately two to three times the maximum VeDBA of the original model – was required to estimate oxygen consumption during feeding. Nevertheless, our results on fold changes of energy cost during feeding behavior corresponded well with the previously reported values, suggesting that video-based VeDBA is a robust method for estimating the significantly higher energy cost of planktivorous fish feeding.

### Energetic cost–benefit balance

**Fig. 3.**
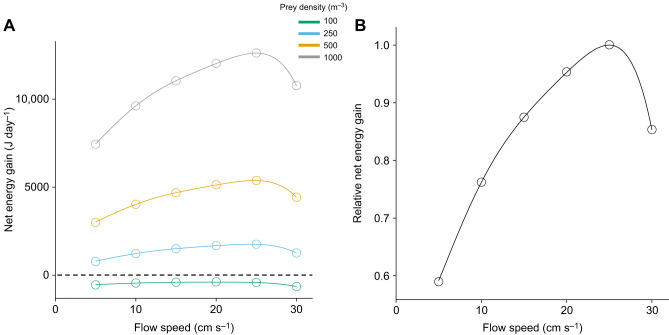
**Net energy gain of *C. viridis*.** The solid lines show (A) the absolute net energy gain with different prey densities in different colors and (B) relative net energy gain at a prey density of 1000 m^−3^. The dashed line in A shows zero net energy gain. Data points are means among individuals (*n*=5).

Using the measured feeding rate and oxygen consumption rate during both steady swimming and feeding, we calculated energy gain and cost per day, assuming 12 h of active feeding and 12 h of continuous swimming with a constant prey density of 1000 m^−3^ (Fig. 3A). Relative net energy gain was also computed by setting the maximum net energy gain as 1 (Fig. 3B). With the threshold of relative net energy gain of 0.9, the energetically efficient range of flow speed for the species was 16.5–29.3 cm s^−1^ (Fig. 3B). Based on past observations (Holzman et al., 2005; Khrizman et al., 2018; Kingsford and MacDiarmid, 1988), a prey density of 1000 m^−3^ should be considered high. Therefore, we further estimated the cost–benefit balance at prey densities of 100, 250 and 500 m^−3^, assuming that feeding rate is a linear function of prey density (Fig. 3A; Ishikawa et al., 2022; Kiflawi and Genin, 1997). The results indicated that the fish can gain excess energy at prey densities >250 m^−3^, but costs surpass gains regardless of the flow speed when prey density is 100 m^−3^ (Fig. 3A).

The range of flow speeds found to be energetically favorable for *C. viridis*, a pectoral fin swimmer, covers intermediate to high flow speeds observed in coral reefs. This finding supports previous studies suggesting that pectoral fin swimmers are well adapted to faster flow environments (Finelli et al., 2009; Fulton and Bellwood, 2005; Marcoux and Korsmeyer, 2019; Schakmann and Korsmeyer, 2023). Our flow measurements indicate that fish experienced this range of flow speeds about a quarter of the time in their habitat, and much weaker flows are found over nearby sandy environments, where no *C. viridis* were observed (Fig. S1). When considering energy cost alone, slower flows are energetically preferred, as faster flows require higher energy expenditure, which is disadvantageous for fish. In terms of energy gain, in contrast, slow flows are not necessarily advantageous as fish capture less prey compared with faster flows with a higher prey flux. In the case of *C. viridis*, energy gain peaked at 25 cm s^−1^ and decreased at faster flows. The net energy gain showed a similar pattern to that of the energy gain as the contribution of energy cost was relatively small compared with energy gain, especially at high prey densities. This result corroborates results from previous studies on river fishes, which also show a relatively small contribution of energy cost to net energy gain (Fausch, 1984; Grossman, 2014; Hill and Grossman, 1993).

Our results showed that the zero net energy gain occurs between prey densities of 100 and 250 m^−3^ (Fig. 3A). To understand the critical relationship between prey density and flow speed above which fish can obtain surplus energy, we further estimated their combination at an energy balance (Fig. S2). The results suggest that when the prey density is 130–160 m^−3^, flow speed plays a significant role that determines whether the net energy gain exceeds zero. The daytime zooplankton density in coral reefs exhibits significant fluctuations, ranging from less than 50 m^−3^ to greater than 2000 m^−3^ (Heidelberg et al., 2004; Noda et al., 1992; Yahel et al., 2005). Therefore, the damselfish may choose faster flow habitats so that they can have a surplus net energy gain even under this low prey density range.

Although fish spent a quarter of their time in a cost-effective range of flow speed, they spent 33.3% of their time at 5–10 cm s^−1^ and 21.8% at 10–15 cm s^−1^ (Fig. 1), which is suboptimal to maximize net energy intake. This inconsistency may result from limitations of our methods. Although we examined effects of unidirectional flows, oscillatory flows significantly increase oxygen consumption rates of reef fish (Marcoux and Korsmeyer, 2019; Schakmann and Korsmeyer, 2023). Amplitude and frequency of oscillatory flows affect fish maneuvers and energy cost. Our field measurements indicate that the fish habitat is dominated by wave-induced oscillatory flows, with frequencies of 0.1–0.5 Hz (Fig. S3), which may require fish to engage in more costly swimming maneuvers than unidirectional flows do. Another potential caveat of our model is the assumption of 12 h of steady swimming during nighttime. This assumption was made to account for the fish's continuous fin flapping motions even at higher frequencies while sheltering within coral branches at night (Goldshmid et al., 2004); however, the energy consumption of these locomotory behaviors within coral branches might differ from that of steady swimming. Also, we used *Artemia* nauplii to eliminate possible effects of changes in prey movements in response to flows. In nature, however, the fish feed on copepods and other marine zooplankton with stronger escape behavior, which may affect foraging performance (Clarke et al., 2005). In addition, incorporation of the energy content of each prey species of the fish's diet composition could further improve the accuracy of the model.

In addition to energetic factors, non-energetic factors also contribute to habitat use and adaptations of fish. For example, the presence of predators and the availability of shelter can significantly impact overall fitness (Grand and Dill, 1997). Fitness of *C. viridis*, a site-attached fish that relies on *Acropola* corals for sheltering, is likely affected by both predators and shelter availability. Interspecific competition can also affect habitat selection. In coral reefs, many species depend on zooplankton, particularly copepods, as a primary food source (Hiatt and Strasburg, 1960). This dietary overlap suggests potential competition for food resources among zooplanktivorous fishes, although fine-scale dietary partitioning might help reduce such competitions (Leray et al., 2019). By incorporating these factors that directly or indirectly affect energy balance, more accurate estimate of habitat use may be possible in future studies.

By combining measurements of oxygen consumption and feeding rate across flow speeds, we empirically estimated the energetic cost–benefit balance of a site-attached zooplanktivorous damselfish in coral reefs. The estimated range of flow speeds at which fish efficiently acquire energy aligned well with those observed in their natural habitats, although the 1-month flow measurement period may not fully capture the long-term hydrodynamic variability of the environment. Despite these limitations, our findings can be applied to estimate habitat use and to assess environmental quality, which may guide conservation efforts in the face of present coastal development around coral reefs.

## Supplementary Material

10.1242/jexbio.251164_sup1 Supplementary information
