# Supplementary material for: Energy costs and benefits of locomotion and feeding in site-attached damselfish
Source: J Exp Biol. 2025 Oct 13;228(19):jeb251164. doi: 10.1242/jeb.251164 (PMC12579945; doi:10.1242/jeb.251164)
Supplement: Supplementary information [file jexbio-228-251164-s1.pdf]

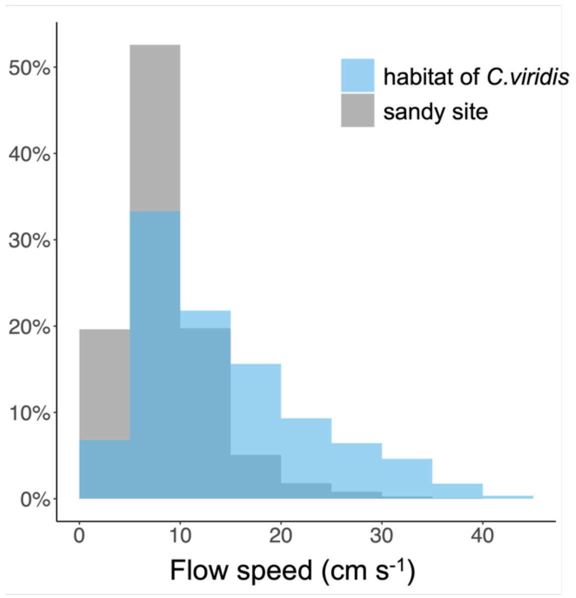

Fig. S1. Comparison of Frequency distribution of flow speeds between a habitat of *C. viridis* and a sandy site. Flow speed was measured by ADV for 10 min every 30 min and averaged over each 10 min measurement. Note that no *C. viridis* was observed in the sandy site.

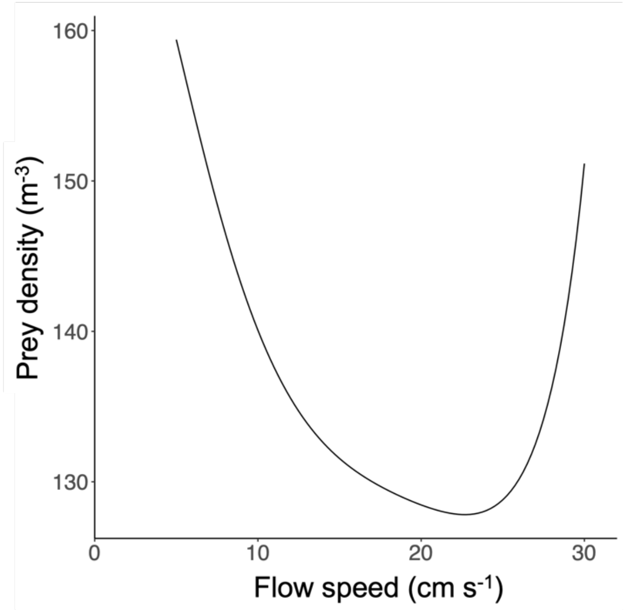

Fig. S2. Relationship between prey density and flow speed above which energy gain surpluses cost.

Journal of Experimental Biology • Supplementary information

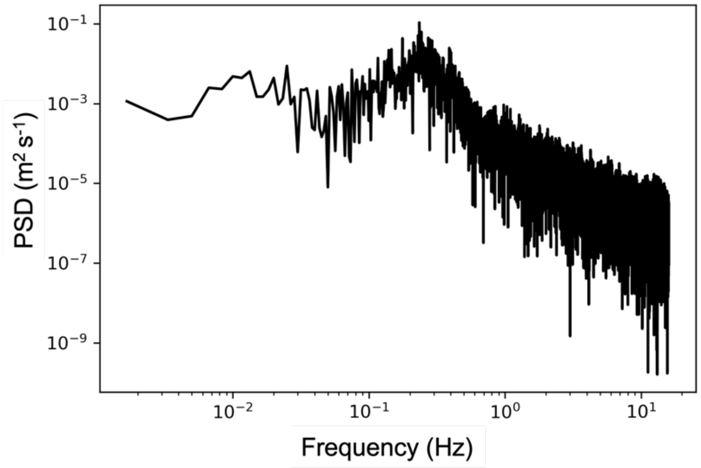

Fig. S3. Representative power spectral density (PSD) of vertical velocity in the fish habitat. PSD was computed from a 10-min long vertical velocity time series data. Wave peak was identified around frequency of 0.1–0.5 Hz.

Journal of Experimental Biology • Supplementary information
